# An unprecedented amplification of near-infrared emission in a Bodipy derived π-system by stress or gelation†
†Electronic supplementary information (ESI) available: Experimental details, synthesis procedures and characterization data of the compounds, and additional figures. CCDC 1523313. For ESI and crystallographic data in CIF or other electronic format see DOI: 10.1039/c7sc01696d



**DOI:** 10.1039/c7sc01696d

**Published:** 2017-06-08

**Authors:** Sandeep Cherumukkil, Samrat Ghosh, Vakayil K. Praveen, Ayyappanpillai Ajayaghosh

**Affiliations:** a Photosciences and Photonics Section , Chemical Sciences and Technology Division , CSIR-National Institute for Interdisciplinary Science and Technology (CSIR-NIIST) , Thiruvananthapuram-695019 , India . Email: ajayaghosh@niist.res.in; b Academy of Scientific and Innovative Research (AcSIR) , CSIR-NIIST Campus , Thiruvananthapuram-695019 , India

## Abstract

We report an unprecedented strategy to generate and amplify near-infrared (NIR) emission in an organic chromophore by mechanical stress or gelation pathways. A greenish-yellow emitting film of π-extended **Bodipy-1**, obtained from *n*-decane, became orange-red upon mechanical shearing, with a 15-fold enhancement in NIR emission at 738 nm. Alternatively, a DMSO gel of **Bodipy-1** exhibited a 7-fold enhancement in NIR emission at 748 nm with a change in emission color from yellow to orange-red upon drying. The reason for the amplified NIR emission in both cases is established from the difference in chromophore packing, by single crystal analysis of a model compound (**Bodipy-2**), which also exhibited a near identical emission spectrum with red to NIR emission (742 nm). Comparison of the emission features and WAXS and FT-IR data of the sheared *n*-decane film and the DMSO xerogel with the single crystal data supports a head-to-tail slipped arrangement driven by the N–H···F–B bonding in the sheared or xerogel states, which facilitates strong exciton coupling and the resultant NIR emission.

## Abstract 2

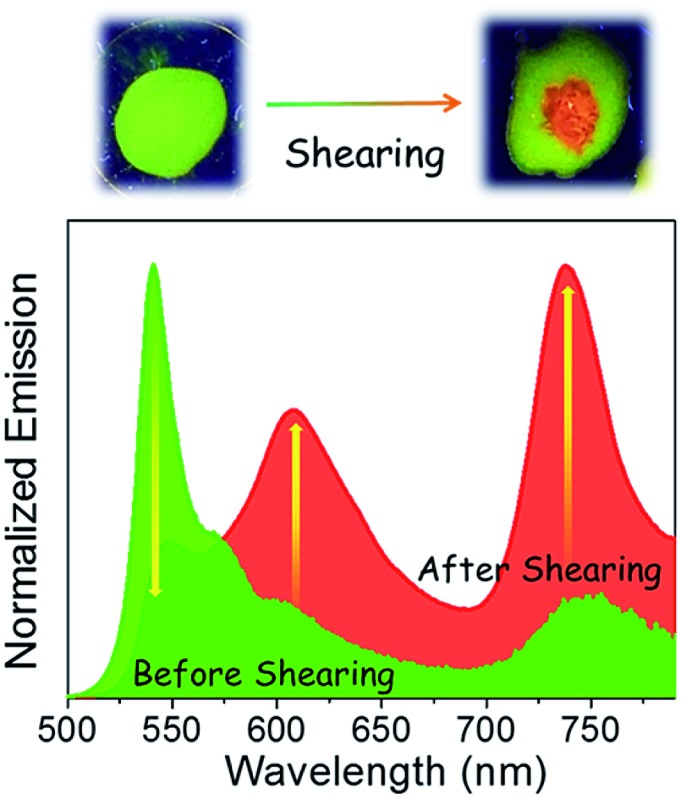
A *meso*-substituted Bodipy derived π-gelator exhibits amplified near-infrared (NIR) emission upon shearing of its film from *n*-decane or drying of its gel from DMSO.

## Introduction

NIR emitting small organic molecules are relatively rare when compared to UV-vis light emitting molecules, however they are important in the fields of materials and biology.^1–3^ For example, NIR emitting chromophores are required for telecommunications, security applications, displays, bio-imaging, *etc.* ^1–3^ Usually, the quantum yield of NIR emission and the stability of the NIR emitting organic molecules are relatively weak.^1,2^ NIR emission is generally achieved by decreasing the HOMO–LUMO gap using strong donor–acceptor interactions, by the extension of π-conjugation or through metal complexation.^1,4^ Molecular self-assembly, as well as the gelation of chromophores, is an alternate approach for the modulation of emission towards longer wavelengths.^5^ In addition, mechanical stress is known to induce modulation of the emission, however in most cases, the modulation occurs in the UV-vis range.^6,7^ In this context, there is a report pertaining to the mechanochromic change of NIR emission to blue emission, and another on mechanically induced phosphorescence in organometallic systems.^8^ However, there are no reports available to date on the stress or gelation induced amplification of NIR emission in organic molecular assemblies.

**Fig. 1:**
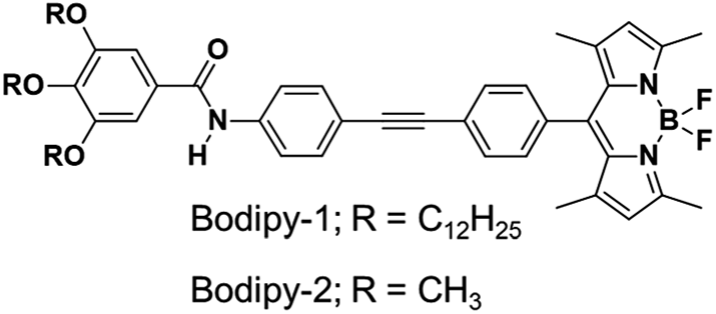
Chemical structures of the Bodipy derivatives under study.

4,4-Difluoro-4-bora-3*a*-4*a*-diaza-*s*-indacene (Bodipy) is a well-studied functional dye due to its intriguing emission properties.^3,9^ The strong and sensitive fluorescence of Bodipy derivatives has been reported to be useful for chemosensing, energy transfer and related optoelectronic applications.^3,9,10^ In addition, self-assembled Bodipy is of interest in the design of soft materials with tunable optical properties and liquid crystalline behavior.^9,11,12^ Herein, we report a previously unknown property of a π-extended Bodipy derivative (Fig. 1) that exhibits amplified NIR emission under mechanical stress or by solvent specific gelation.

The *meso*-phenyleneethynylene substituted Bodipy derivatives, **Bodipy-1** and **Bodipy-2**, were synthesized by Sonogashira coupling between 5,5-difluoro-10-(4-iodophenyl)-1,3,7,9-tetramethyl-5*H*-dipyrrolo[1,2-*c*:2′,1′-*f*][1,3,2]diazaborinin-4-ium-5-uide (3) and 3,4,5-tris(alkoxy)*N*-(4-ethynylphenyl)-benzamide (9)^11*f*^ (Schemes S1–S3†). The detailed synthetic procedures and characterization are discussed in the ESI.†

In chloroform (1 × 10^–4^ M), **Bodipy-1** exists in the monomeric state with absorption maxima at 315 and 504 nm with a shoulder band at 474 nm (Fig. S1†). The absorption maximum at 315 nm corresponds to the phenyleneethynylene part, whereas the narrow absorption maximum at 504 nm (*ε* = 88 400 M^–1^ cm^–1^) corresponds to the strong S_0_–S_1_ electronic transition involving the (0 → 0) vibrational states of Bodipy.^11^ The broad absorption feature observed around 380 nm can be assigned to the S_0_–S_2_ transition of the Bodipy unit.^11^ The high fluorescence quantum yield (*Φ*_f_ = 0.53, *λ*_ex_ = 475 nm, fluorescein in 0.1 M NaOH as standard, *Φ*_f_ = 0.91) and the small Stokes shift (608 cm^–1^) observed are indications of the singlet emitting excited state (Fig. S1†). The identical quantum yields of 0.52 and 0.53 at two different excitation wavelengths, 315 nm (corresponding to phenyleneethynylene) and 474 nm (corresponding to Bodipy), indicated good electronic communication between the two moieties.

**Fig. 2:**
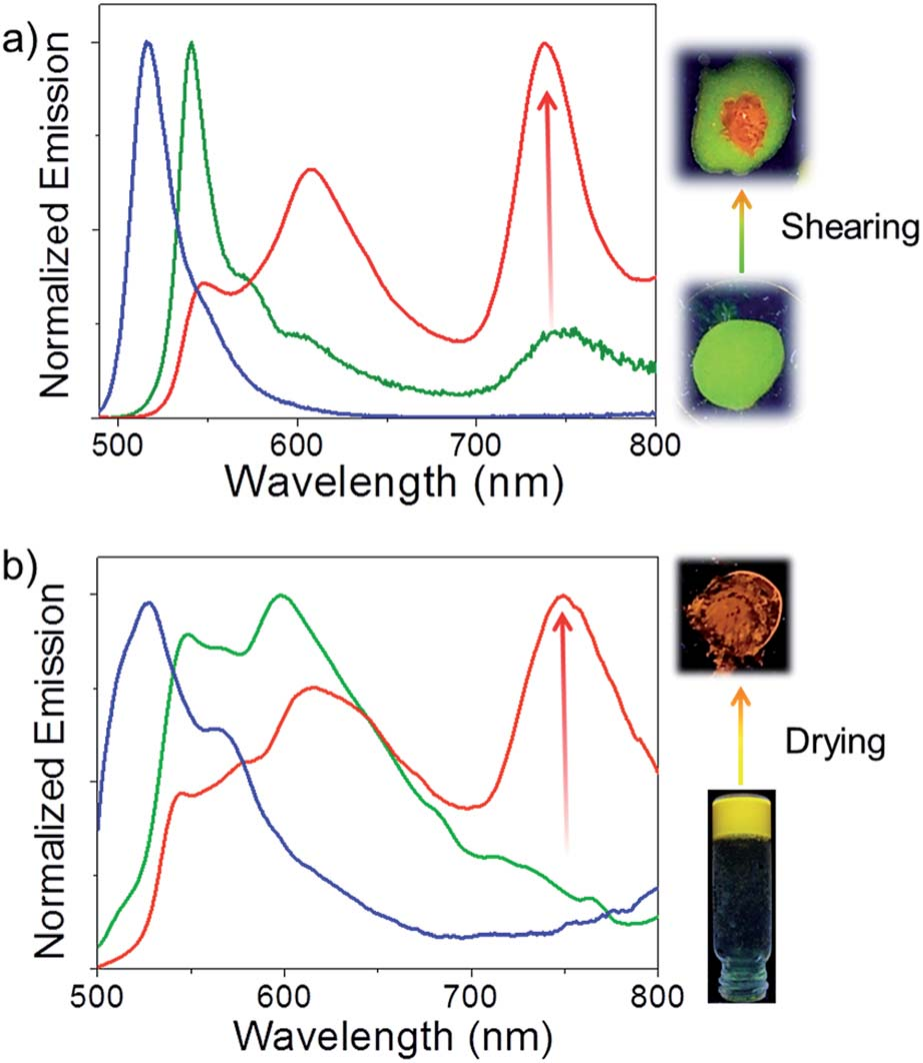
(a) Emission profiles of **Bodipy-1** self-assembly in *n*-decane (1 × 10^–4^ M, blue); film processed from *n*-decane self-assembly (green) and after shearing the film (red), *λ*_ex_ = 475 nm. (b) Emission profiles of **Bodipy-1** self-assembly in DMSO solution (1 × 10^–4^ M, blue), DMSO gel (green) and xerogel (red), *λ*_ex_ = 475 nm. The corresponding fluorescence color changes under UV light illumination are also shown.

Comparison of the optical properties of **Bodipy-1** in a variety of solvents (1 × 10^–4^ M, Fig. S2 and S3†) reveals an additional red-shifted band at 530 nm in *n*-decane, probably due to J-type aggregation.^11*e*^ The variable temperature absorption spectral change in *n*-decane indicates a decrease in the intensity of the shoulder band at 530 nm (Fig. S3†). The emission in *n*-decane solution occurred at 516 nm with a shoulder band at around 542 nm (Fig. 2a). A film prepared from the *n*-decane solution exhibited a greenish-yellow emission with a maximum at 541 nm, and two shoulder bands at 574 and 604 nm (Fig. 2a). Surprisingly, a weak NIR band is observed at 738 nm, which was absent in the *n*-decane solution. The absolute quantum yield (*Φ*_f_) of the film measured by a calibrated integrated sphere attached to the spectrofluorimeter is 0.076 (±0.008). When the film was mechanically sheared, the intensity of the initial emission band at 541 nm decreased, with increases in the emission intensities at 608 and 738 nm (15-fold, Fig. 2a) without much change in the absorption spectrum (Fig. S4†). The emission color of the sheared portion of the film changed to orange-red from the initial greenish-yellow (Fig. 2a, inset) with a fluorescence quantum yield (*Φ*_f_) of 0.079 (±0.008). Reversibility of the mechanochromic emission changes is possible by re-aggregating the sheared sample in *n*-decane (Fig. S5†).

In DMSO (1 × 10^–4^ M), the molecule exhibited a broad absorption spectrum with maxima at 310 and 511 nm (Fig. S3a†). The emission spectrum shows a maximum at 527 nm with a shoulder at 566 nm (Fig. 2b). Interestingly, in DMSO at a concentration of 1 × 10^–2^ M, a gel was formed which exhibited a yellow emission (Fig. 2b and Fig. S6†). The emission spectrum of the DMSO gel shows a broad band between 500 and 800 nm, with two maxima at 548 and 598 nm with a weak shoulder between 700 and 800 nm. Surprisingly, when the gel was transferred onto a glass plate followed by evaporation of the solvent, an orange-red emission was observed (Fig. 2b, inset). In this process, the intensities of the initial emission peaks at 548 and 598 nm significantly decreased and a strong NIR emission band at 748 nm appeared with 7-fold enhancement. The emission spectrum of the DMSO xerogel of **Bodipy-1** is identical to that of the mechanically sheared *n*-decane film (Fig. 2), indicating the possibility that in the sheared film and xerogel states, the molecular packing may be identical. This hypothesis was further supported by comparing the fluorescence decay profiles of the sheared *n*-decane film with that of the DMSO xerogel. For this experiment, both films were excited with 375 nm light and the emission was collected at 606 and 740 nm (Fig. S7 and Table S1†). The sheared *n*-decane film exhibited a triexponential decay (*λ*_em_ = 740 nm) with lifetimes of 0.31 (45.37%), 1.43 (34.93%) and 2.16 ns (19.7%). Interestingly, the xerogel also exhibited a triexponential decay (*λ*_em_ = 740 nm) with near identical lifetimes of 0.39 (48.6%), 1.0 (41.2%) and 2.74 ns (10.2%), confirming the presence of similar molecular aggregates. This observation was further confirmed by monitoring the emission decay at 606 nm. Furthermore, the absence of long lifetime components when the emission was monitored at 740 nm rules out the possibility of any excimer formation. These observations indicate that the sharp NIR emission originates from a slipped molecular organization, analogous to previous reports on the spectral properties of Bodipy systems.^11e,13^ In order to support the above argument, a detailed study of the molecular packing was conducted by single crystal, film state wide angle X-ray scattering (WAXS) and attenuated total reflection (ATR) FT-IR analyses.

**Fig. 3:**
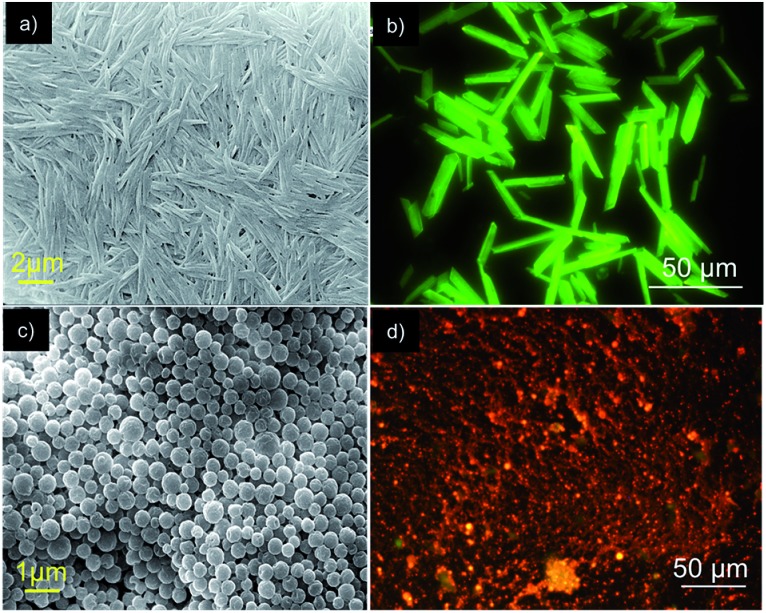
(a and c) SEM images and (b and d) fluorescence microscopy images of **Bodipy-1** self-assembly in *n*-decane (1 × 10^–4^ M) and xerogel, respectively.

Before going into the details of the exact molecular packing of **Bodipy-1** self-assembly, we attempted to understand the morphological features. Fluorescence microscopy images of the assembly in *n*-decane revealed the formation of greenish-yellow emitting rods (Fig. 3b). The formation of these micro-rods was confirmed by scanning electron microscopy (SEM) analysis, where the rods were found to have a maximum length of 50 μm and a thickness of less than 5 μm, as seen in the SEM images (Fig. 3a and S8†). Interestingly, micrometer sized spherical particles were observed under SEM for the DMSO xerogel (Fig. 3c). This morphology is an exception to the usually observed fibrous morphology of organogels.^5*c*^ The fluorescence microscopy image of the xerogel revealed that these particles are orange emitting (Fig. 3d).

**Fig. 4:**
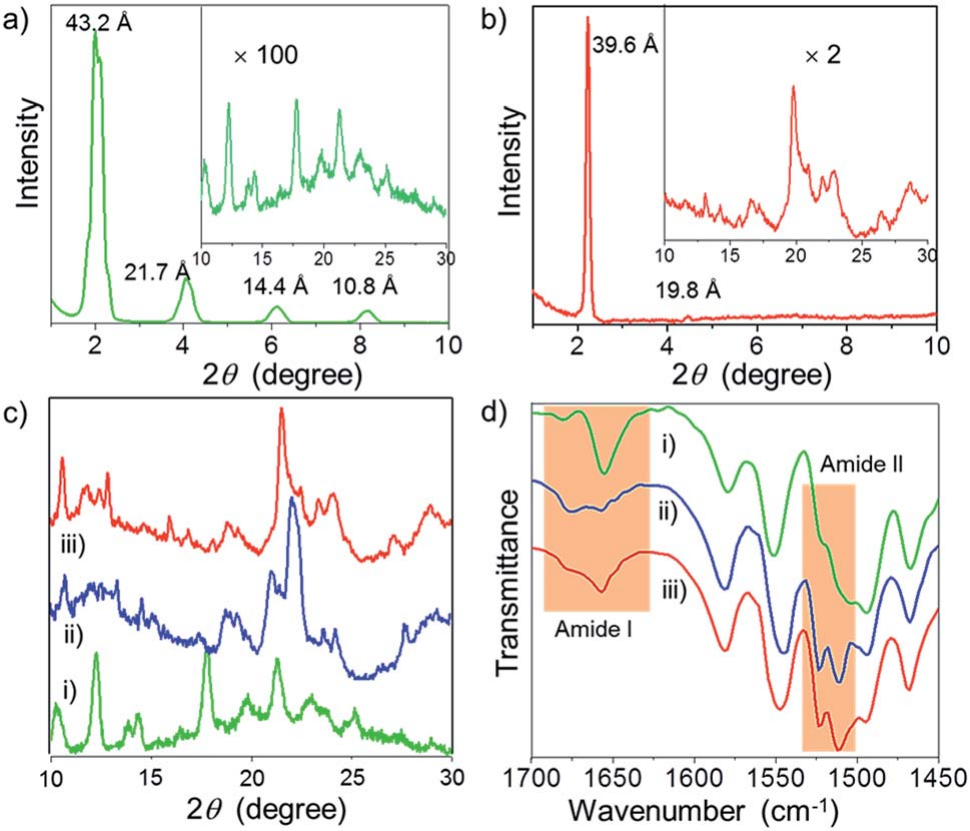
WAXS of **Bodipy-1** (a) film processed from *n*-decane and (b) xerogel. (c) Comparison of WAXS (2*θ* = 10–30°) and (d) FT-IR (ATR) of: (i) the *n*-decane film, (ii) the *n*-decane film after shearing and (iii) the DMSO xerogel.

For an in-depth understanding of the molecular interactions and packing of **Bodipy-1** in the *n*-decane film, before and after shearing and also in the DMSO xerogel, WAXS experiments were performed. WAXS revealed the formation of a sharp crystalline lamellar assembly in *n*-decane with a *d*-spacing of 43.2 Å, having a reciprocal spacing ratio of 1 : 2 : 3 : 4 (Fig. 4a).^7b,14^ In addition, several weak signals were seen in the range of 2*θ* = 10–30°, indicating a lesser extent of π-stacking (Fig. 4a inset). On the other hand, long range ordering was disturbed upon shearing, as revealed from the disappearance of the sharp peaks at lower 2*θ* values (Fig. S9†). Also, the WAXS pattern of the sheared film indicates reorganization in the short range ordering with respect to the pristine film (Fig. 4c). The DMSO xerogel exhibited a lamellar organization (Fig. 4b) with a sharp peak at 2*θ* = 2.25° (*d*-spacing = 39.6 Å).^7b,14^ A comparison of the diffraction peaks of the xerogel with the *n*-decane film before and after shearing at the wide-angle region of 10–30° is shown in Fig. 4c. These data revealed a considerable similarity in the molecular packing of the xerogel and the sheared *n*-decane film.

**Fig. 5:**
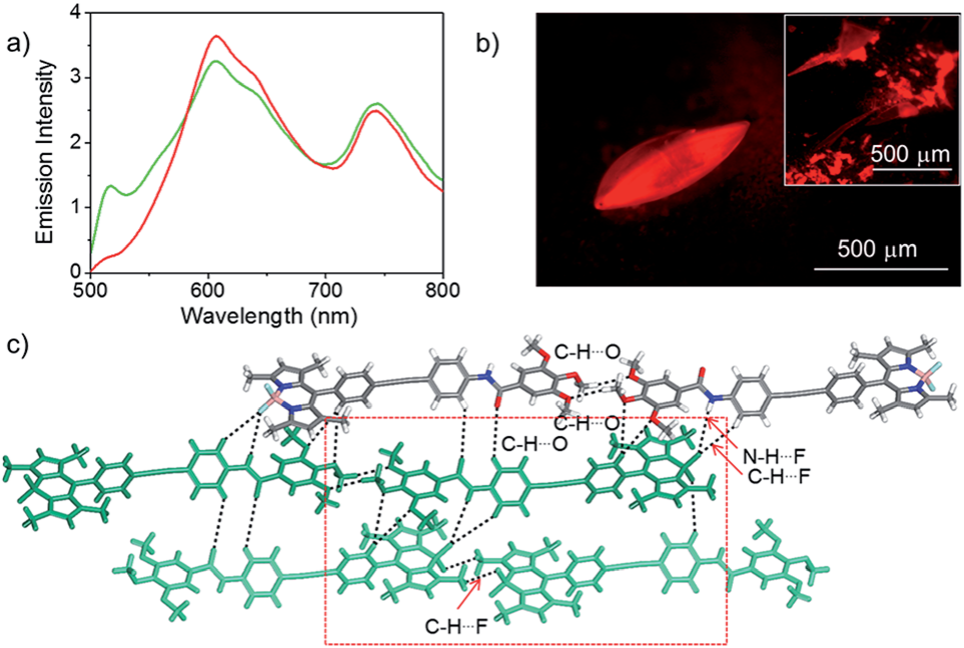
(a) Emission spectrum before (red) and after (green) shearing, (b) the fluorescence microscopy image and (c) crystal packing with different interactions of the **Bodipy-2** single crystal. The inset shows the emission of the sheared crystal. The red square shows Bodipy–Bodipy interaction.

In order to gain more insight into the molecular organization in the assembly, before and after shearing and also in the xerogel, we carried out single crystal X-ray analysis of a model derivative of **Bodipy-1**. For this purpose, **Bodipy-2** was synthesized and single crystals were grown from a chloroform/*n*-hexane solvent mixture by the vapor diffusion method. The **Bodipy-2** crystals exhibited a red emission with an NIR band at 742 nm, which did not show any further change upon grinding, indicating a stable molecular packing (Fig. 5 and S10†). The emission profile of the **Bodipy-2** single crystal (Fig. 5a) was similar to that of the **Bodipy-1** xerogel (Fig. 2b) except for the slight variation in the intensities of the peaks, indicating identical molecular packing in both cases.

**Fig. 6:**
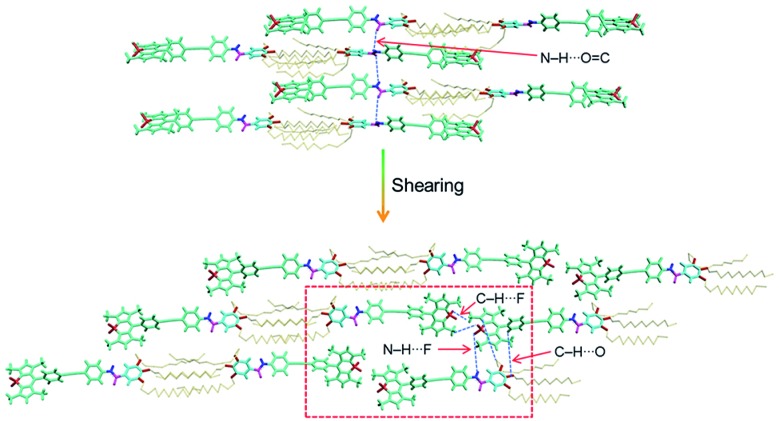
Molecular organization of **Bodipy-1** before and after shearing of the film processed from *n*-decane. The red square highlights the Bodipy–Bodipy interaction responsible for NIR emission as in the single crystal (Fig. 5c).

**Bodipy-2** (*P*1-space group) has a slipped-stack packing, as is evident from the crystal structure analysis (Fig. 5c and S11†). The molecules form extended chains through distinct centric C–H···O_(–OMe)_ and C–H···F_(Bodipy)_ interactions. These chains stack to yield a centrosymmetric head-to-tail arrangement of the chromophores. In a typical head-to-tail arrangement, the observed N–H···F bonds^16*a*^ are augmented by a range of weak interactions such as C–H···O, C–H···F and C–H···π.^13*c*^ Furthermore, the terminal Bodipy moieties of a unique dimer form π–π interactions with the 3,4,5-trisalkoxybenzamide group of the two adjacent dimers. An average stacking distance of 3.64 Å qualifies for an optimized stacking interaction. The interactions are quantified by Hirshfeld surface and two-dimensional fingerprint analyses of the crystal structure.^16*b*^ In the absence of any classic hydrogen bonds, the structure is dominated by a wide variety of weak interactions such as C–H···π (23.4%), C–H···O (11.1%), N–H···F (7.9%) and other non-covalent interactions (Fig. S12 and S13†). In order to confirm that the molecular packing in the sheared *n*-decane film as well as the DMSO xerogel of **Bodipy-1** is comparable to that of the single crystal of **Bodipy-2**, the WAXS pattern of the former is compared with the simulated powder X-ray diffraction peaks of the latter (Fig. S14†). These data indicate more or less identical structural organization in both cases. Therefore, it is reasonable to propose an extended molecular organization in the *n*-decane film, which upon shearing changes into a more organized assembly as shown in Fig. 6.

FT-IR (ATR) spectral data of **Bodipy-1** (Fig. 4d) indicate the presence of intermolecular H-bonds either with the carbonyl oxygen (C=O) or with the fluorine attached to the boron (B–F).^16*a*^ The FT-IR spectrum of the *n*-decane film before shearing revealed H-bonded N–H stretching frequencies at 3320 (major) and 3421 cm^–1^ (Fig. S15†), indicating 1-D supramolecular polymer formation through amide H-bonding.^11f,14,15^ Shearing resulted in the rupture of the amide H-bonding which is clear from the appearance of the 3421 cm^–1^ band.

To gain a deeper insight, we monitored the carbonyl stretching vibrations around 1650 cm^–1^ (amide I) and N–H bending vibrations around 1530 cm^–1^ (amide II).^14^ The sharp amide I band at 1656 cm^–1^ gets broadened with an equally intense band at 1676 cm^–1^ upon shearing (Fig. 4d). This result indicates that shearing leads to the partial breakage of the H-bond between N–H and C=O. Consequently, the N–H moiety must be H-bonded to the B–F moiety upon shearing, which is supported by the presence of a sharp band at 1523 cm^–1^. The FT-IR spectrum of the DMSO xerogel very much resembles that of the sheared *n*-decane film (Fig. 4d).

The reason for the observed NIR emission in **Bodipy-2** is obvious from its crystal packing data, which can also be extended to the **Bodipy-1** xerogel and sheared film states. The multiple H-bonding and conformational rigidity in the crystal and xerogel may lead to strong exciton coupling, resulting in low energy NIR emission. Many other Bodipy derivatives have been shown to have red and NIR emissions under different conditions.^13^ In a recent report, Yamamoto and co-workers have shown that, in Bodipy-polystyrene microspheres, energy migration within different aggregates of Bodipy is responsible for the observed low-energy red and NIR emissions.^13*f*^ Analogous to the above reports and from the crystal structure, WAXS, FT-IR and photophysical data, it can be concluded that **Bodipy-1** forms ordered aggregates with low-optical band gaps similar to those in **Bodipy-2** crystals, which upon excitation generate NIR emission (Fig. 5c and 6). As in the case of our previous reports with oligo(*p*-phenylenevinylene) based gels,^17^ it is possible that **Bodipy-1** forms aggregates of different energy levels, emitting at different long wavelengths, as indicated by the broad emission spectrum. The population of such higher-order aggregates responsible for the low-energy emissions can be manipulated by applying stress, which eventually results in the amplification of the NIR emission.

## Conclusions

In conclusion, presented herein is a unique approach to generate NIR emission in an organic chromophore. Even though mechanical stress and gelation have been previously shown to modulate the emission of organic chromophores, this is the first demonstration of the stimuli induced amplified generation of NIR emission. Moreover, **Bodipy-1** is the first π-gelator which exhibits NIR emission in the xerogel state. The *meso*-π-extended molecular structure of **Bodipy-1**, the weak π-stacking between chromophores, and the labile supramolecular amide linkage facilitate the mechanical stress induced formation of a head-to-tail extended slipped molecular packing that allows strong electronic coupling between the chromophores. This report may encourage further studies on Bodipy and related chromophores to generate controlled NIR emission for specific applications.
