# Supplementary material for: An unprecedented amplification of near-infrared emission in a Bodipy derived π-system by stress or gelation
Source: Chem Sci. 2017 Jun 8;8(8):5644–9. doi: 10.1039/c7sc01696d (PMC5621002; doi:10.1039/c7sc01696d)
Supplement: Supplementary file 1 [file SC-008-C7SC01696D-s001.pdf]

Electronic Supplementary Material (ESI) for Chemical Science.
This journal is © The Royal Society of Chemistry 2017

Supplementary Information for

An Unprecedented Amplification of Near-Infrared Emission in a Bodipy Derived π-System by Stress or Gelation

Sandeep Cherumukkil,[a,b] Samrat Ghosh,[a,b] Vakayil K. Praveen, [a,b] and Ayyappanpillai Ajayaghosh* [a,b]

[a] Photosciences and Photonics Section, Chemical Sciences and Technology Division, CSIR-National Institute for Interdisciplinary Science and Technology (CSIR-NIIST), Thiruvananthapuram-695019, India, E-mail: ajayaghosh@niist.res.in

[b] Academy of Scientific and Innovative Research (AcSIR), CSIR-NIIST Campus, Thiruvananthapuram-695019, India

Index

1. Materials and Methods S3
    1.1. Synthesis-General Procedures S3
    1.2. Synthesis-Characterization Techniques S3
    1.3. Measurements S3
    1.4. Scheme for the Synthesis S4
2. Supplementary Information Table and Figures S10
    Fig. S1 and S2 . . . . S10
    Fig. S3 and S4 . . . . S11
    Fig. S5 and Table S1 . . . . S12
    Fig. S6 and S7 . . . . S13
    Fig. S8 and S9 . . . . S14
    Fig. S10 and S11 . . . . S15
    Fig. S12 and S13 . . . . S16
    Fig. S14 and S15 . . . . S17
3. Supplementary Information References S18

1. Materials and Methods

1.1. Synthesis – General Procedures

Unless otherwise stated, all starting materials and reagents were purchased from commercial suppliers and used without further purification. The solvents used for the reactions were purified and dried by employing the standard laboratory methods. The reactions were monitored using thin layer chromatography on silica gel 60 F_{254} (0.2 mm; Merck) or Al_2O_3 (0.2 mm; Merck) and visualization was accomplished using a hand held UV-lamp (365 nm). Column chromatography was employed to purify the reaction products using glass column packed with silica gel of size range 100- 200 microns.

1.2. Synthesis – Characterization Techniques

NMR spectra were measured on a 300 or 500 MHz Bruker Avance DPX spectrometer. Chemical shifts are reported in parts per million (ppm) using tetramethylsilane (TMS) (δ_H = 0 ppm) as an internal reference. The resonance multiplicity is described as *s* (singlet), *d* (doublet), *t* (triplet) and *m* (multiplet). FT-IR spectra were recorded on a Shimadzu IR Prestige-21 Fourier Transform Infrared Spectrophotometer (FT-IR) using KBr pellet method. Electrospray ionization (ESI) high-resolution mass spectra were recorded on using Thermo Scientific Exactive mass spectrometer. Matrix-assisted laser desorption ionization time-of-flight (MALDI-TOF) mass spectra were obtained on a Shimadzu AXIMA-CFR PLUS spectrometer using α-cyano-4-hydroxycinnamic acid as the matrix.

1.3. Measurements

The electronic absorption spectra were recorded on a Shimadzu spectrophotometer UV-2100. The fluorescence spectra were recorded on a SPEX-Fluorolog-3 FL3-221 spectrofluorimeter. Solid-state emission spectra were recorded using a front face sample holder. Optical studies in solution-state were carried out in a 1 cm quartz cuvette. Relative quantum yield measurements were carried out at two different excitation wavelengths (315 and 475 nm) for the monomeric state using fluorescein in 0.1 N NaOH as the standard which has a reported quantum yield of Φ_f = 0.91. Absolute fluorescence quantum yields in the film state were measured by using a calibrated integrating sphere attached to SPEX Fluorolog spectrofluorimeter and the quantum yield value was estimated based on the de Mello method.[S1] Lifetime measurements were carried out using IBH (model 5000 DPS) time-correlated single photon counting (TCSPC) system. Experiments were carried out with 375 nm LED excitation source (pulse width < 100 ps, repetition rate 1 MHz). Film state FT-IR spectra recorded on a Shimadzu IR Prestige-21 FT-IR spectrophotometer using attenuated total reflection (ATR)-8200HA accessory. SEM images were taken on a Zeiss EVO 18 cryo

SEM Special Edn with variable pressure detector working at 20-30 kV after sputtering with gold. Samples were prepared by drop cast the aggregates of Bodipy-1 in *n*-decane and DMSO gel over a smooth aluminium foil. The drop cast samples were allowed to dry under normal condition for one day and subsequently subjected to drying under vacuum for 12 h. Fluorescence microscopy images were taken by using Leica DFC 290 microscope. The WAXS analysis was carried out using XEUSS SAXS/WAXS system by Xenocs, operated at 50 kV and 0.6 mA. The data were collected in the transmission mode geometry using Cu-K_α radiation of wavelength 1.54 Å. The single crystal analysis of the model derivative, Bodipy-2 was carried out on a Bruker SMART APEX CCD diffractometer with graphite-monochromatized Mo-*K*, λ = 0.71073 Å radiation.

1.4. Scheme for the Synthesis

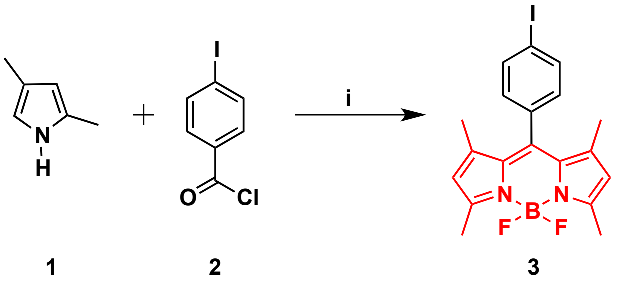

Scheme S1. Reagents and conditions: **i**) a) Dry dichloroethane, reflux, 3 h; b) Et_3N, BF_3•OEt_2, 25 °C then reflux, for 30 min, 36%.[S2]

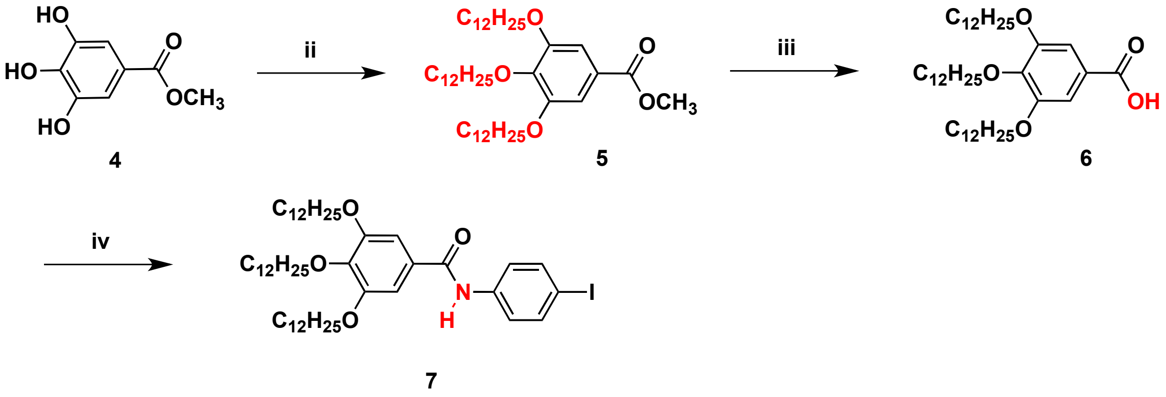

Scheme S2. Reagents and conditions: **ii**) 1-Bromododecane, K_2CO_3, DMF, 100 °C, 24 h, 78%; **iii**) KOH, ethanol, reflux, 12 h, 80%; **iv**) a) $SOCl_2$, dry dichloromethane, rt., 5 h; b) 4-Iodoaniline, dry toluene, rt., 12 h, 50%.[S3]

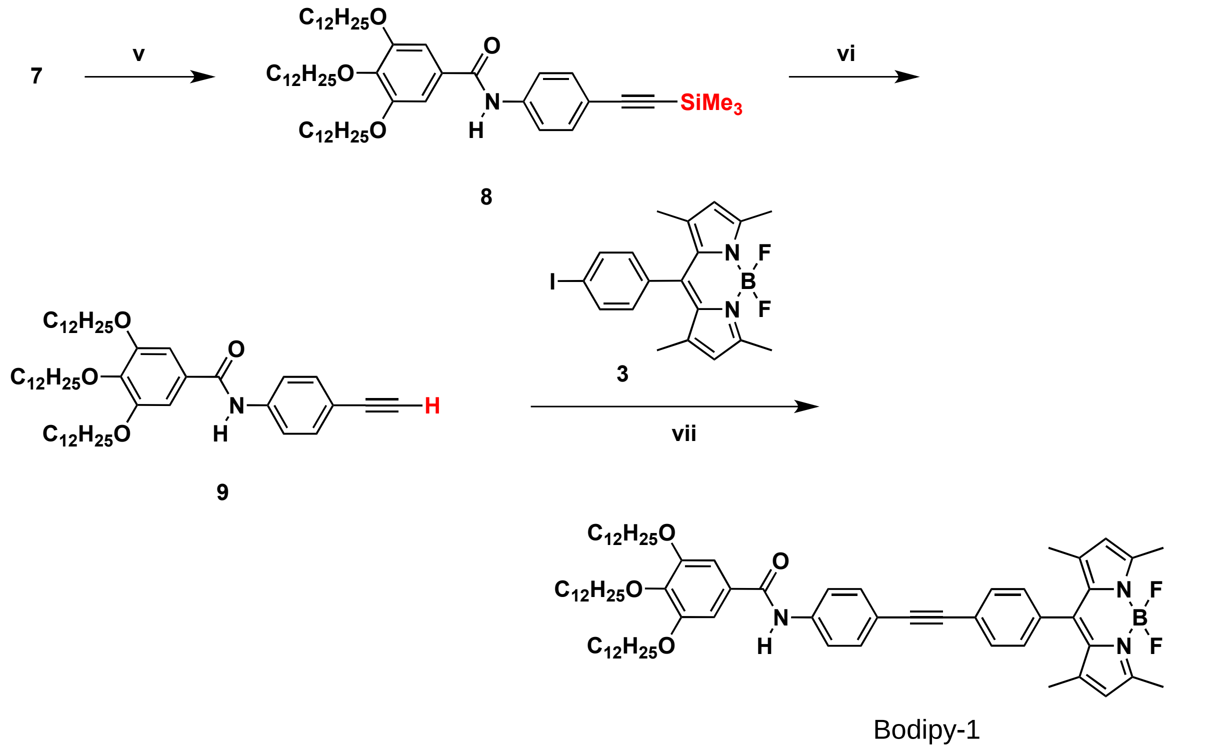

Scheme S3. Reagents and conditions: **v**) Trimethylsilyl acetylene, CuI, $PdCl_2(PPh_3)_2$, dry Et_3N, dry THF (oxygen free), rt., 12 h, 80%; **vi**) KF, dichloromethane/methanol (1:5), rt, 12 h, 76%;[s3] **vii**) CuI, $PdCl_2(PPh_3)_2$, dry Et_3N, dry THF (oxygen free), 60 °C, 24 h, 30%.

Preparation of 3,4,5-tris(dodecyloxy)-N-(4-((trimethylsilyl)ethynyl)phenyl)benzamide (8)[S3]

Compound **7** (3.0 mmol), bis(triphenylphosphine)palladium (II) dichloride (10 mol%), and copper (I) iodide (10 mol%) were added to an oven-dried two-neck round bottom flask equipped with a magnetic stirring bar. The round bottom flask was then sealed with a rubber septum, evacuated and backfilled with argon three times. Degassed triethylamine (10 ml) was added followed by degassed THF (20 ml). After stirring for 5 minutes at room temperature, trimethylsilyl acetylene (4.45 mmol) was added dropwise into the reaction mixture and continued stirring for 12 h. The reaction mixture was extracted using chloroform and washed with dilute hydrochloric acid. The organic layer was washed with brine and dried over anhydrous sodium sulphate and then evaporated under reduced pressure. The residue thus obtained purified by silica gel column chromatography using 2% ethylacetate-*n*-hexane as an eluent. Yield: 80%, ^{1}H NMR (300 MHz, $CDCl_3$): δ = 7.79 (*s*, 1H; -N*H*), 7.59 (*d*, *J* = 9 Hz, 2H; Phenyl-*H*), 7.47 (*d*, *J* = 8.7 Hz, 2H; Phenyl-*H*), 7.02 (*s*, 2H, Phenyl-*H*), 4.01 (*t*, *J* = 6 Hz, 6H; -OC*H*$_2$-), 1.84-1.75 (*m*, 6H; -C*H*$_2$-), 1.5-1.26

(*m*, 54H; -CH_2-), 0.88 (*t*, *J* = 6 Hz, 9H; -CH_3), 0.05 (*s*, 9H; -Si*Me*$_3$) ppm; MALDI-TOF-MS (matrix: α-cyano-4-hydroxycinnamic acid): m/z calculated for $C_{54}H_{91}NO_4Si$ [M+H]$^+$: 846.72, found: 847.0.

Preparation of 3,4,5-tris(dodecyloxy)-N-(4-ethynylphenyl)benzamide (9)[s3]

To a solution of **8** (2.25 mmol) in 10 ml dichloromethane, KF (44.9 mmol) in 50 ml methanol was added to it and allowed to stir at room temperature for 12 h. After completion of the reaction, the reaction mixture was extracted using chloroform, washed with water, brine and then dried over anhydrous sodium sulphate. Solvent was evaporated under reduced pressure. The residue thus obtained purified by silica gel column chromatography using 5% ethylacetate/*n*-hexane as an eluent afforded the compound **9** as a white solid. Yield: 76%; ^{1}H NMR (300 MHz, $CDCl_3$): δ = 7.79 (*s*, 1H; -N*H*), 7.61 (*d*, *J* = 8.5 Hz, 2H; Phenyl-*H*), 7.50 (*d*, *J* = 8.4 Hz, 2H; Phenyl-*H*), 7.02 (*s*, 2H; Phenyl-*H*), 4.02 (*t*, *J* = 6 Hz, 6H; -OCH_2-), 3.07 (*s*, 1H; Acetylenic-*H*), 1.84-1.75 (*m*, 6H; -CH_2-), 1.5-1.26 (*m*, 54H; -CH_2-), 0.87 (*t*, *J* = 6 Hz, 9H; -CH_3) ppm; MALDI-TOF-MS (matrix: α-cyano-4-hydroxycinnamic acid): m/z calculated for $C_{51}H_{83}NO_4$ [M+H]$^+$: 774.61, found: 774.6.

Preparation of Bodipy-1 and Bodipy-2

Compound **3** (0.47 mmol), bis(triphenylphosphine)palladium (II) dichloride (10 mol%), and copper (I) iodide (10 mol%) were added to an oven-dried two-neck round bottom flask equipped with a magnetic stirring bar. The round bottom flask was then sealed with a rubber septum, evacuated and backfilled with argon three times. Degassed triethylamine (30 ml) was added followed by degassed THF (50 ml). After stirring for 5 minutes at room temperature, compound **9** (0.57 mmol) dissolved in 10 ml (1:1) mixture of degassed triethylamine and THF was added and the reaction mixture was stirred at 60 °C for 24 h. The reaction mixture was extracted using chloroform and washed with dilute hydrochloric acid. The organic layer was washed with brine and dried over anhydrous sodium sulphate and then evaporated under reduced pressure. The crude product was then purified by column chromatography using silica gel as adsorbent. The pure compound (**Bodipy-1**) was eluted with 40% dichloromethane-*n*-hexane solvent mixture. Yield: 30%; FT-IR (KBr): ν_{max} = 3420, 3325, 3293, 2924, 2852, 2211, 1909, 1657, 1583, 1550, 1522, 1509, 1495, 1468, 1427, 1403, 1390, 1373, 1362, 1334, 1309, 1260, 1237, 1194, 1155, 1117, 1089, 1049, 1020, 984, 899, 871, 832, 816, 764, 719, 707 cm^{-1}; ^{1}H NMR (500 MHz, $CDCl_3$): δ = 7.79 (*s*, 1H; -N*H*), 7.67 (*d*, *J* = 8 Hz, 4H; Phenyl-*H*), 7.57 (*d*, *J* = 8 Hz, 2H; Phenyl-*H*), 7.3 (*d*, *J* = 8 Hz, 2H; Phenyl-*H*), 7.05 (*s*, 2H; Phenyl-*H*), 6.0 (*s*, 2H; =C*H*-), 4.05-4.01 (*m*, 6H; -OCH_2-), 2.56 (*s*, 6H; pyrrole-CH_3), 1.84-1.76 (*m*, 6H; -CH_2-), 1.50-1.47 (*m*, 6H; -CH_2-), 1.44 (*s*, 6H; pyrrole-CH_3), 1.36-1.27 (*m*, 48H; -CH_2-) 0.88 (*t*, *J* = 6.5 Hz, 9H; -CH_3) ppm; ^{13}C NMR (125 MHz, $CDCl_3$): δ = 165.62, 155.75, 153.31, 143.04, 141.77, 140.86, 138.37, 134.85, 133.59,

132.56, 132.25, 131.23, 129.57, 128.23, 124.19, 121.37, 119.80, 118.51, 105.86, 100.00, 90.59, 88.45, 73.61, 69.52, 31.93, 30.33, 29.74, 29.71, 29.67, 29.65, 29.59, 29.41, 29.37, 26.08, 22.70, 14.61, 14.12 ppm; HRMS (ESI): m/z calculated for $C_{70}H_{100}BF_2N_3O_4$ $[M+H]^+$: 1096.78544, found: 1096.78459.

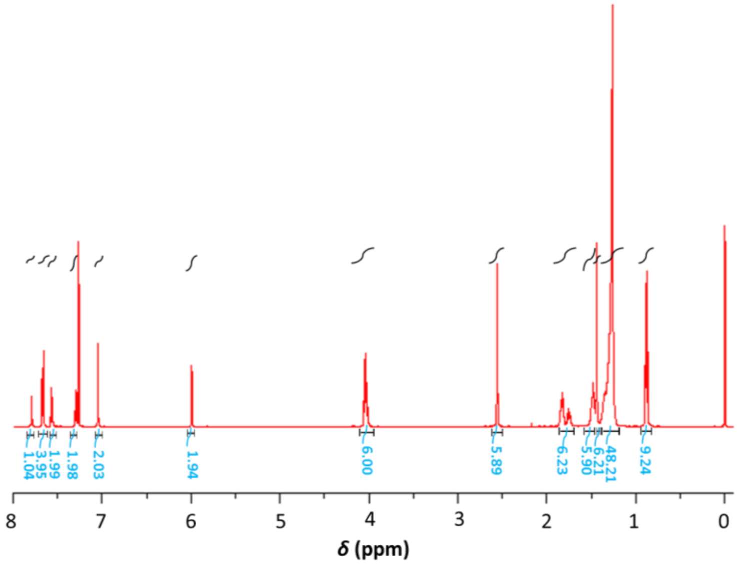

^1H NMR (500 MHz) spectrum of **Bodipy-1** in $CDCl_3$

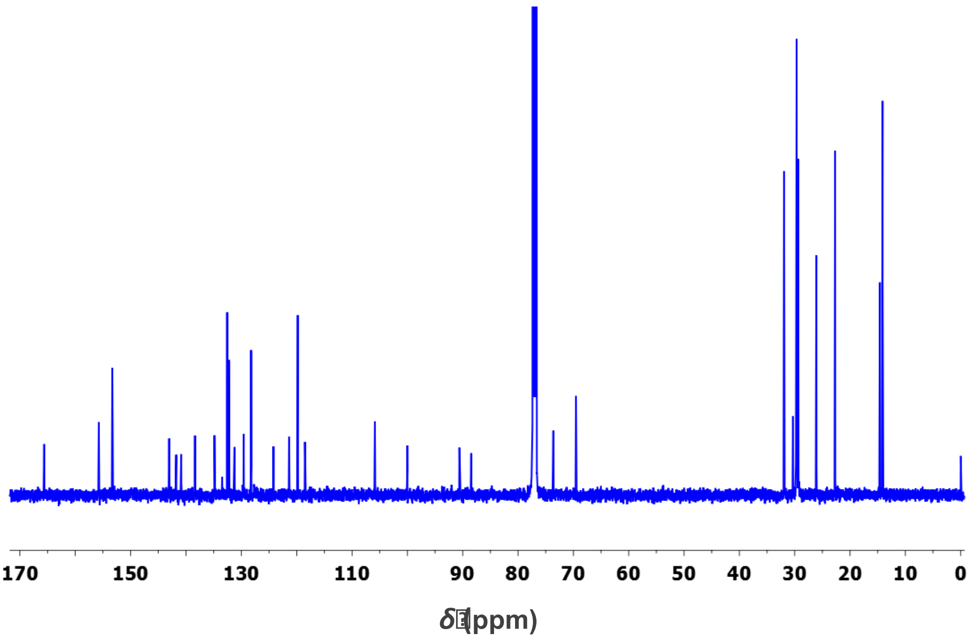

^{13}C NMR (125 MHz) spectrum of **Bodipy-1** in $CDCl_3$

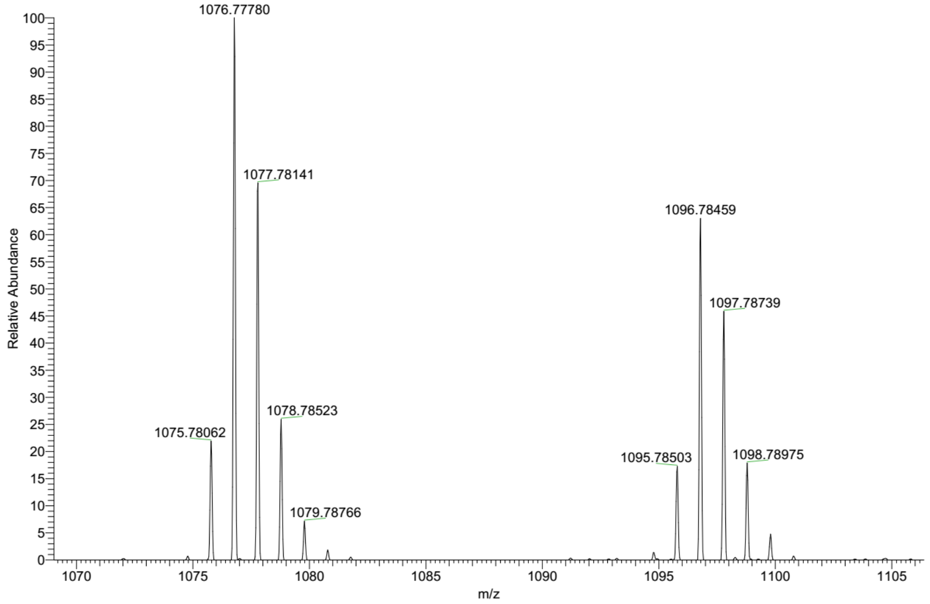

High-resolution mass spectrum (HRMS-ESI) of **Bodipy-1**

Similar synthesis procedure has been adopted for **Bodipy-2**, where the 1-bromododecane is replaced with 1-iodomethane. Yield: 40%; FT-IR (KBr): ν_{max} = 3387, 3000, 2955, 2924, 2853, 2213, 1738, 1676, 1585, 1545, 1514, 1499, 1468, 1412, 1406, 1368,1335, 1308, 1288, 1261, 1240, 1196, 1160, 1128, 1084, 1055, 1001, 986, 972, 870, 851, 837, 831, 816, 766, 737, 704 cm^{-1}; ^{1}H NMR (500 MHz, $CDCl_3$): δ = 7.81 (*s*, 1H; -N*H*), 7.68 (*d*, *J* = 8 Hz, 4H; Phenyl-*H*), 7.58 (*d*, *J* = 8 Hz, 2H; Phenyl-*H*), 7.3 (*d*, *J* = 8 Hz, 2H; Phenyl-*H*), 7.09 (*s*, 2H, Phenyl-*H*), 6.0 (*s*, 2H; =C*H*-), 3.94 (*s*, 9H; -OCH_3), 2.56 (*s*, 6H; pyrrole-CH_3), 1.44 (*s*, 6H; pyrrole-CH_3) ppm; ^{13}C NMR (125 MHz, $CDCl_3$): δ = 165.56, 155.94, 153.64, 143.69, 141.97, 140.93, 138.40, 135.48, 135.11, 132.75, 132.41, 130.26, 128.42, 124.34, 121.53, 120.04, 118.94, 118.25, 104.81, 100.16, 92.82, 91.01, 88.70, 86.93, 61.14, 56.65, 29.51, 14.74, ppm; HRMS (ESI): m/z calculated for $C_{37}H_{34}BF_2N_3O_4$ $[M+Na]^+$: 656.25081, found: 656.25214.

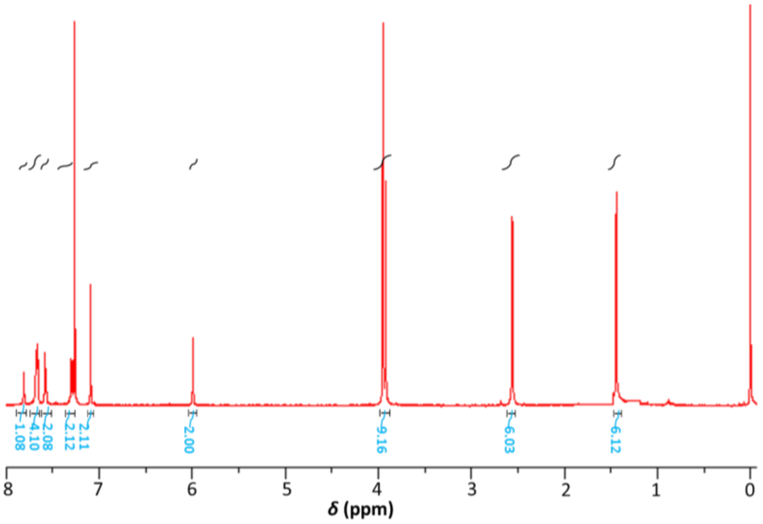

^{1}H NMR (500 MHz) spectrum of **Bodipy-2** in $CDCl_3$

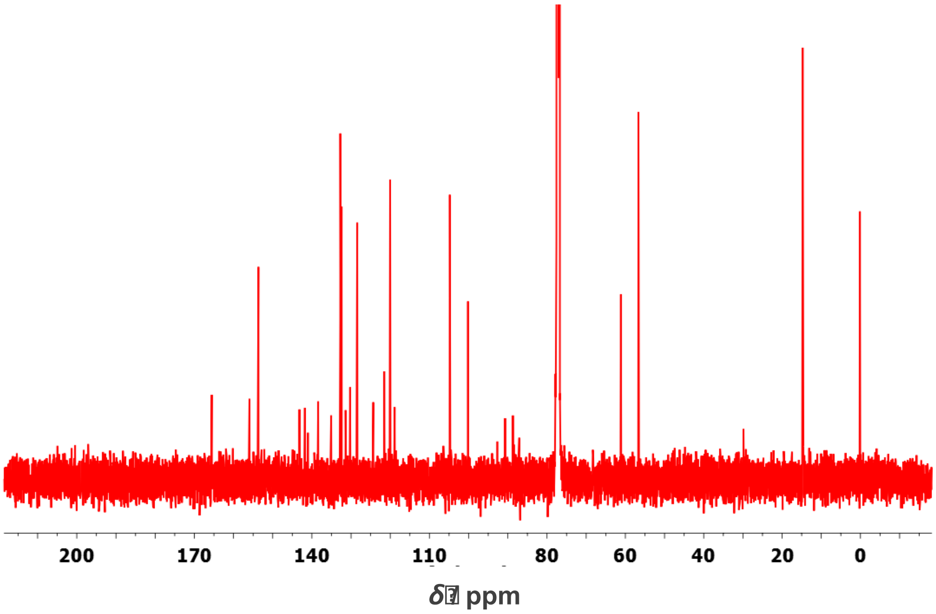

^{13}C NMR (125 MHz) spectrum of **Bodipy-2** in $CDCl_3$

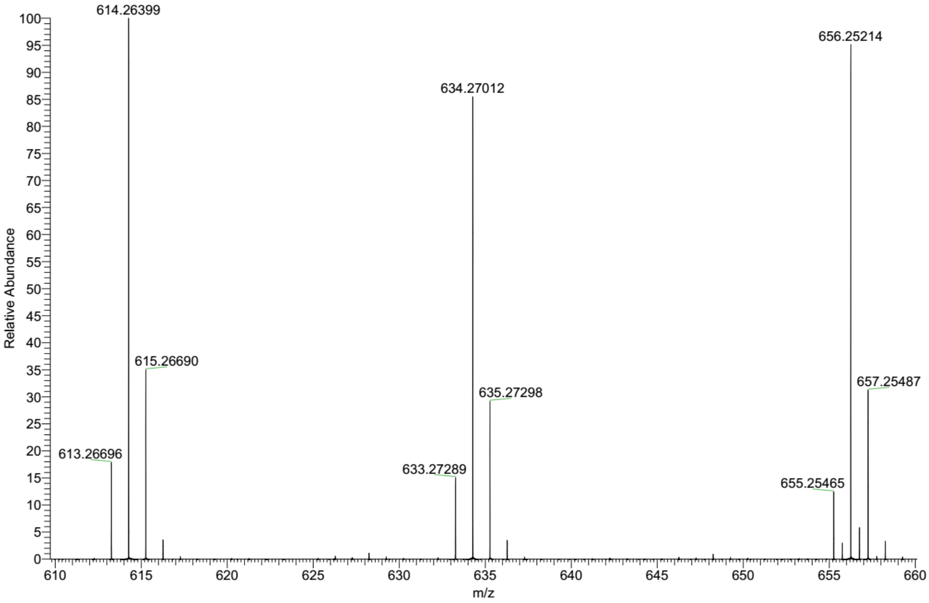

High-resolution mass spectrum (HRMS-ESI) of **Bodipy-2**

2. Supplementary Information Table and Figures

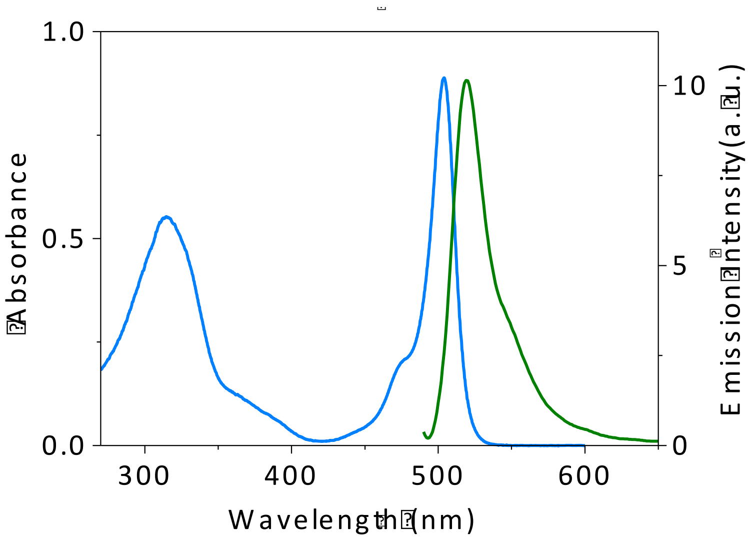

Fig. S1 Absorption (blue) and emission (green) spectra of **Bodipy-1** in chloroform (1 x 10^{-4} M) λ_{ex} = 475 nm.

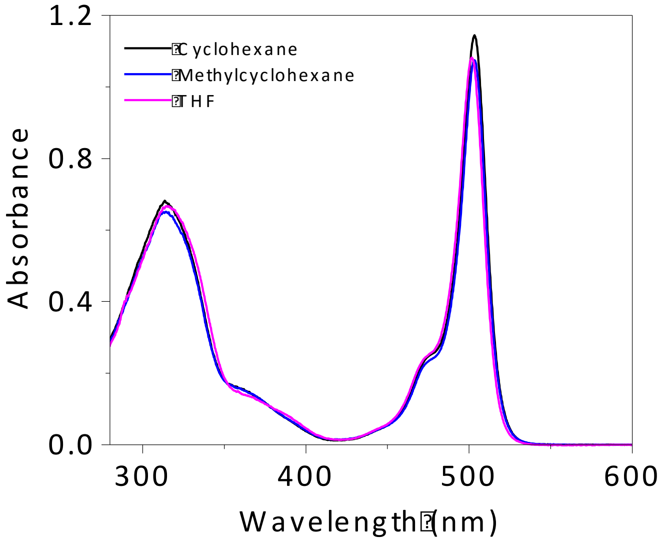

Fig. S2 Absorption spectra of **Bodipy-1** in cyclohexane, methylcyclohexane and THF (c = 1 x 10^{-4} M).

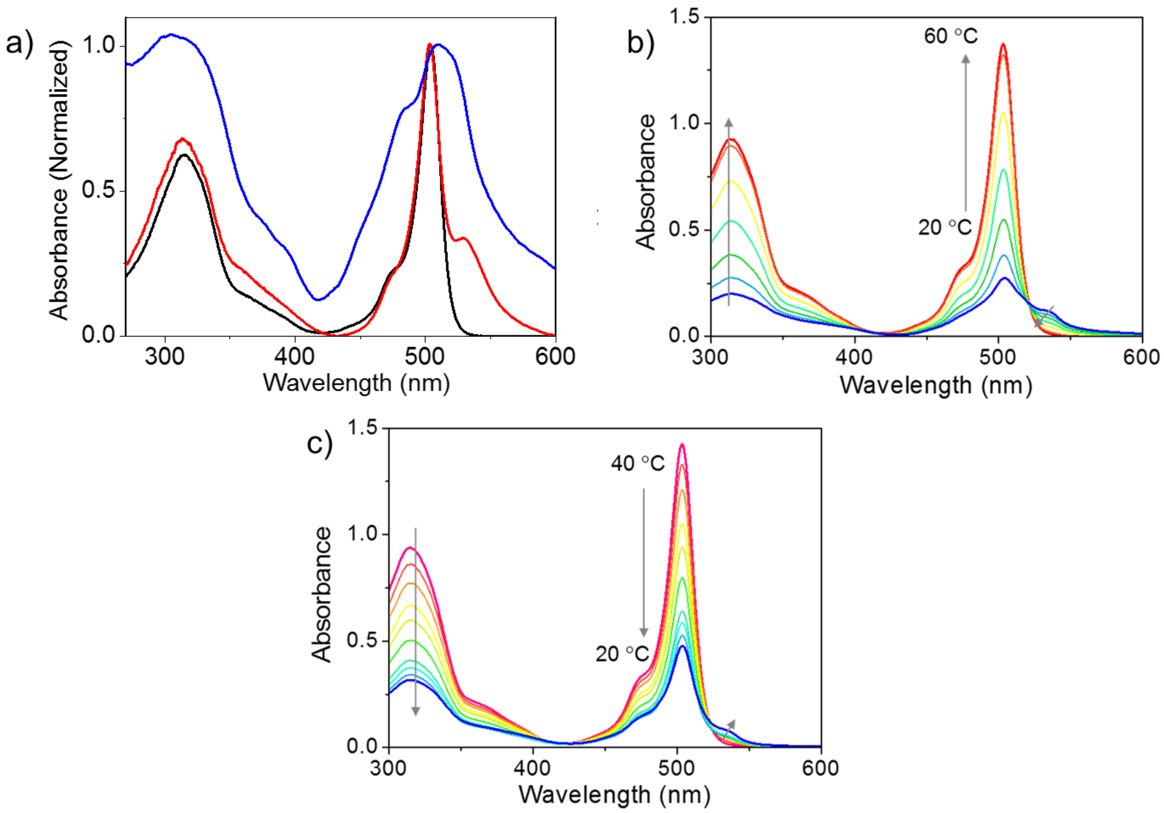

Fig. S3 a) Normalized absorption spectra (c = 1 x 10^{-4} M) of **Bodipy-1** in $CHCl_3$ (black), *n*-decane (red) and DMSO (blue). Temperature-dependent absorption spectra of **Bodipy-1** aggregates in *n*-decane (c = 2 x 10^{-4} M), b) heating and c) cooling.

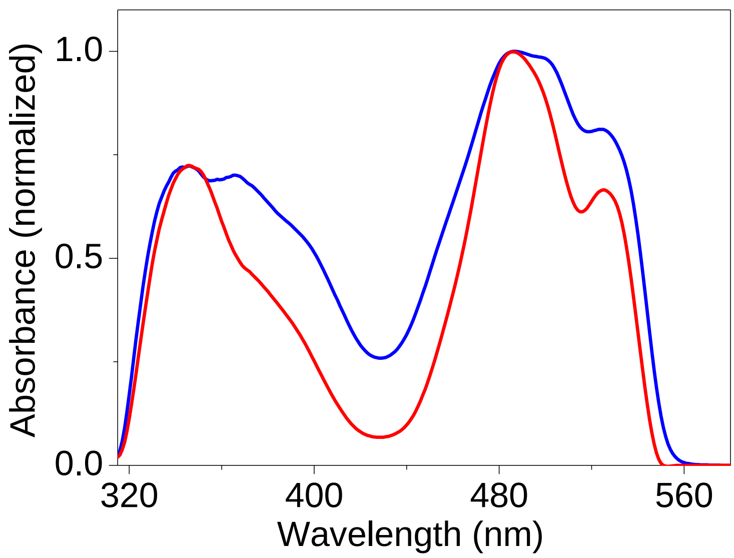

Fig. S4 Absorption spectra of **Bodipy-1** *n*-decane film before (red) and after shearing (blue).

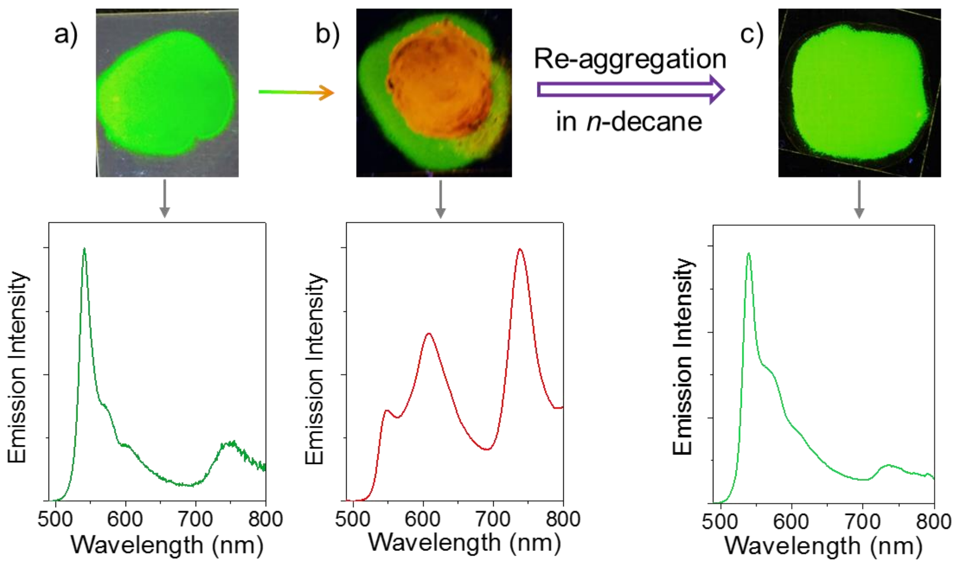

Fig. S5 Photographs and corresponding emission spectra of the *n*-decane film a) before, b) after shearing. c) Sheared sample was dissolved and re-aggregated in *n*-decane and film was prepared via evaporating solvent.

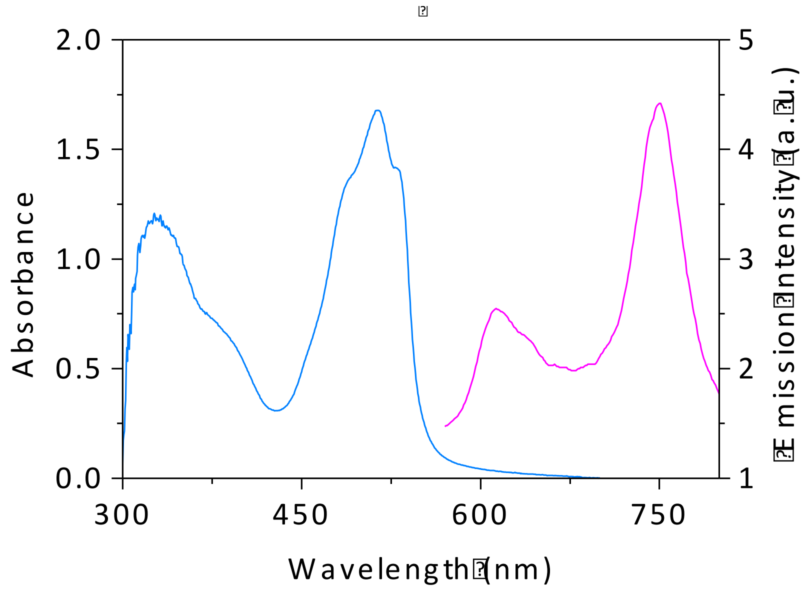

Fig. S6 Absorption and emission spectrum (λ_{ex} = 550 nm) of **Bodipy-1** DMSO xerogel.

Table S1. Fluorescence lifetime data **Bodipy-1** sheared *n*-decane film and DMSO xerogel (λ_{ex} = 375 nm).

**Bodipy-1**	**Sheared *n*-Decane Film**	**DMSO Xerogel**
**Emission monitored at 606 nm**	0.70 (39.26%), 2.13 (52.12%) and 6.1 ns (8.62%)	0.80 (20.3%), 2.48 (58.92%) and 5.70 ns (20.78%)
**Emission monitored at 740 nm (NIR band)**	0.31 (45.37%), 1.43 (34.93%) and 2.16 ns (19.7%).	0.39 (48.6%), 1.0 (41.2%) and 2.74 ns (10.2%)

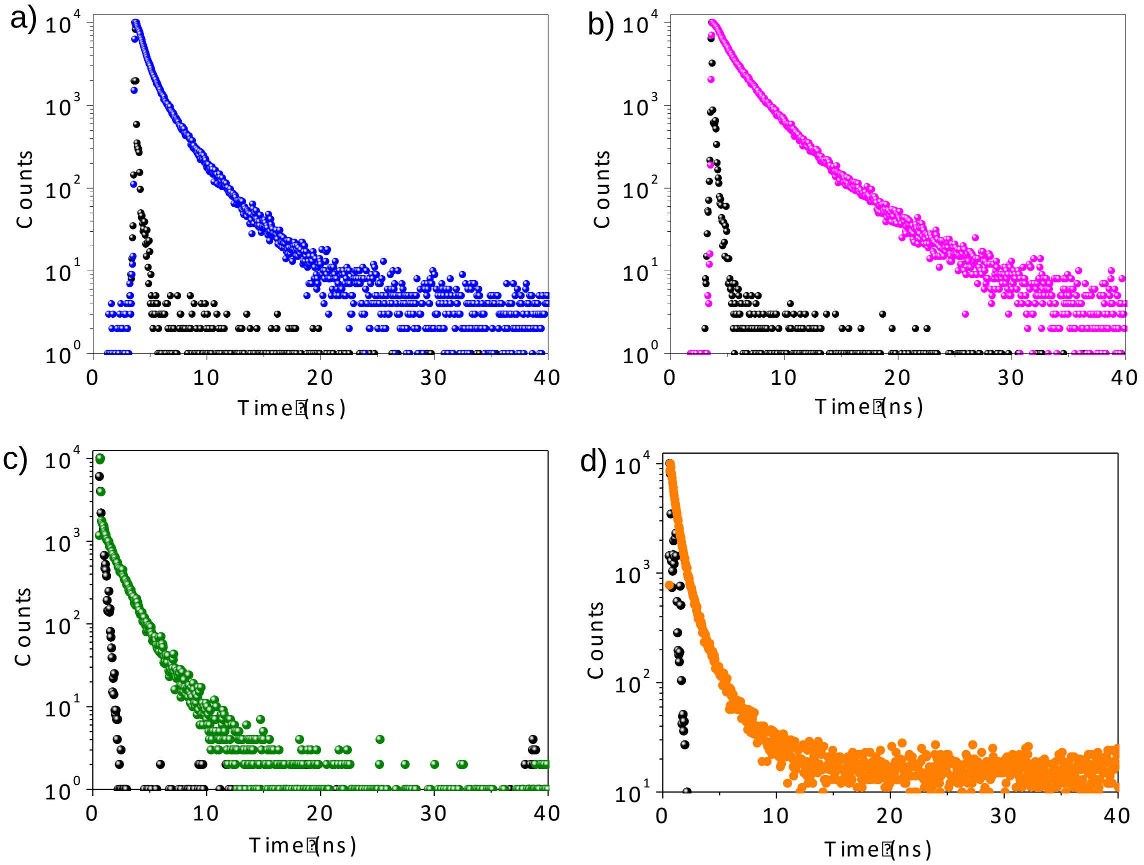

Fig. S7 Lifetime decay profiles of **Bodipy-1** when emission monitored at 606 nm a) sheared *n*-decane film (blue) and b) DMSO xerogel (pink). Lifetime decay profiles of **Bodipy-1** when emission monitored at 740 nm corresponding to NIR band c) sheared *n*-decane film (green) and d) DMSO xerogel (orange). In all cases black profile corresponds to prompt and 375 nm LED used for excitation.

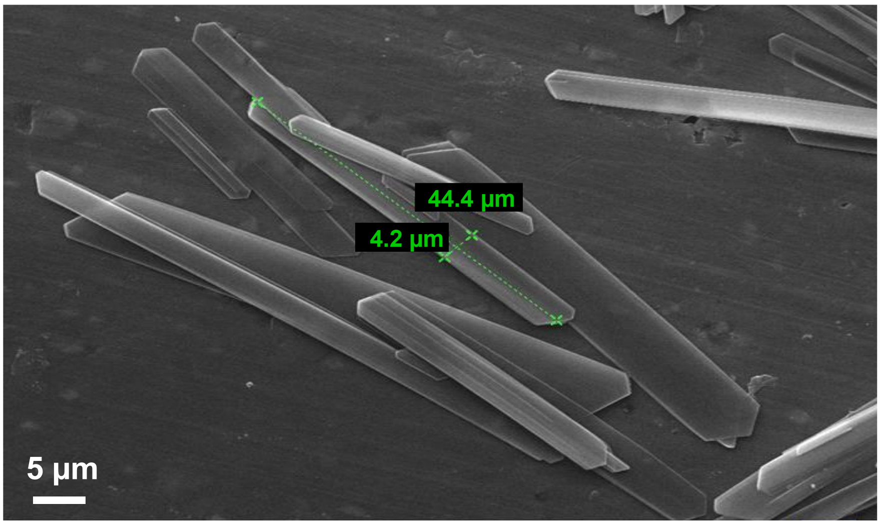

Fig. S8 SEM image of **Bodipy-1** aggregates in *n*-decane (1 x 10^{-4} M).

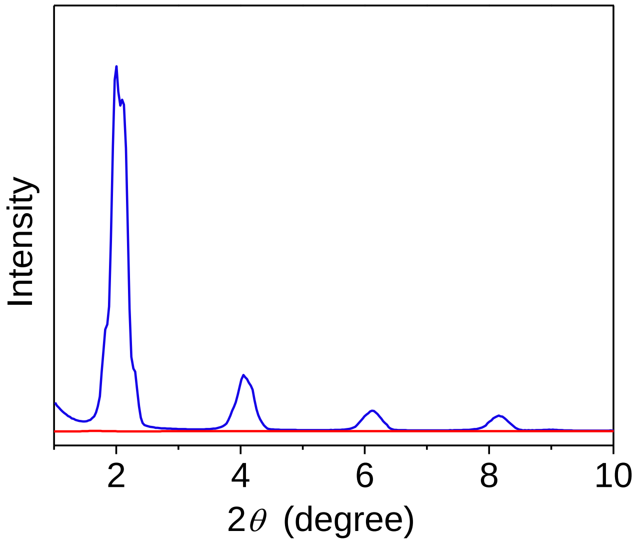

Fig. S9 WAXS of **Bodipy-1** film processed from *n*-decane (blue) and the same film after shearing (red).

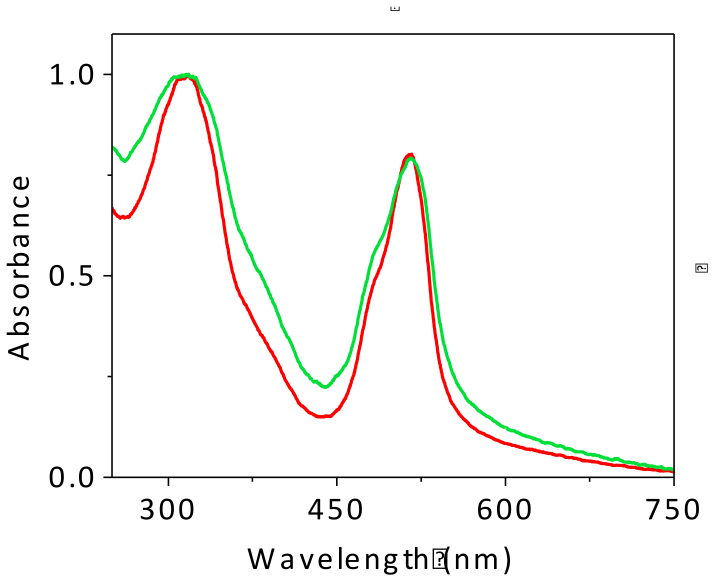

Fig. S10 Absorption spectrum of **Bodipy-2** single crystal before (red) and after (green) shearing.

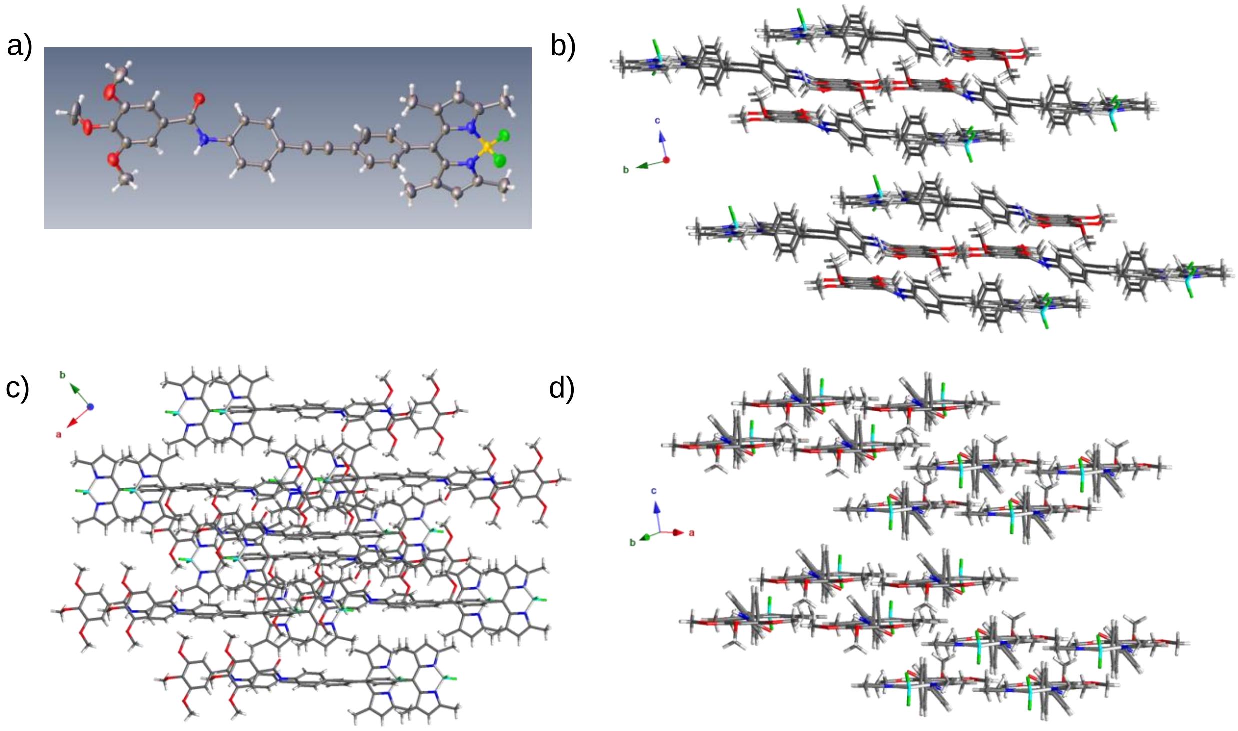

Fig. S11 Bodipy-2 single crystal a) structure b-d) packing viewed from different axes.

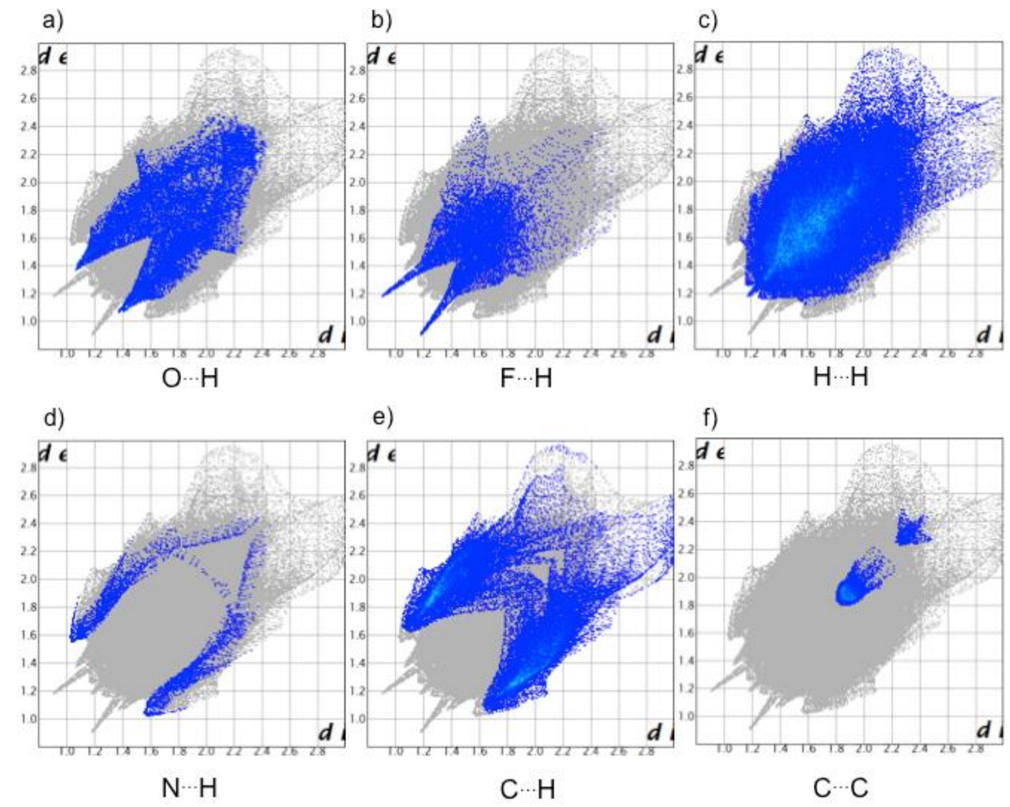

Fig. S12 Hirshfeld 2D-fingerprint plots depicting various interactions in the **Bodipy-2** crystal.[S4] Sharp spikes for F···H, O···H and pair of wings observed for C···H and N···H interactions.

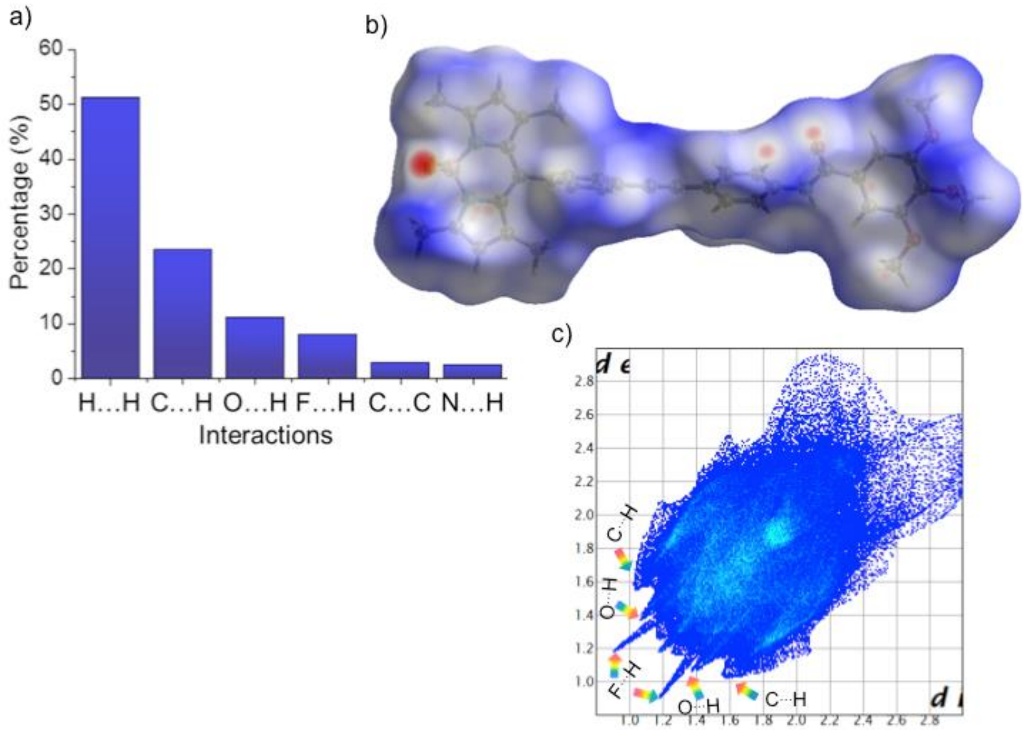

Fig. S13 a) Histogram representing various intermolecular interactions in **Bodipy-2**. b) Hirshfeld surface (d_{norm}) analysis of **Bodipy-2**. c) Hirshfeld 2D-fingerprint plot showing prominent interactions.[S4]

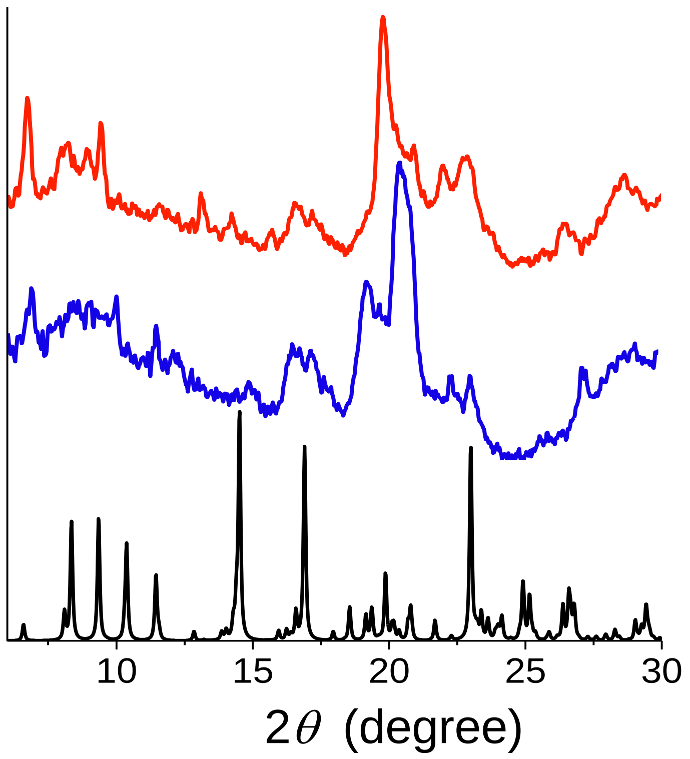

Fig. S14 Simulated WAXS pattern correspond to Bodipy-2 (black) and the WAXS pattern of **Bodipy-1** *n*-decane sheared film (blue) and DMSO xerogel (red).

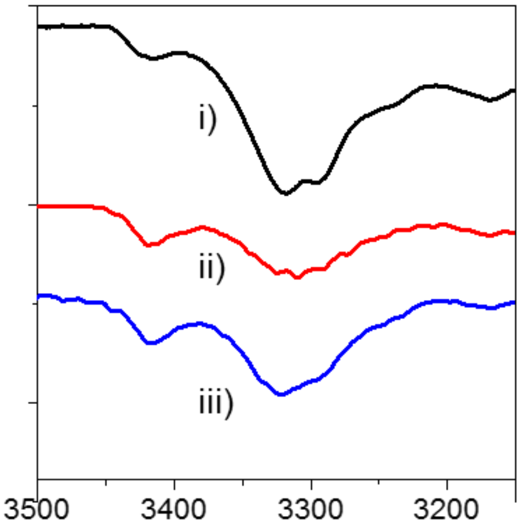

Fig. S15 FT-IR spectra of **Bodipy-1** *n*-decane film i) before and ii) after shearing and iii) DMSO xerogel.

3. Supplementary Information References

S1. J. C. de Mello, H. F. Wittmann and R. T. Friend, *Adv. Mater.*, 1997, **9**, 230–232.

S2. J. Chen, A. Burghart, C.-W. Wan, L. Thai, C. Ortiz, J. Reibenspies and K. Burgess, *Tetrahedron Lett.,* 2000, **41**, 2303–2307.

S3. (*a*) R. Thirumalai, R. D. Mukhopadhyay, V. K. Praveen and A. Ajayaghosh, *Sci. Rep.,* 2015, **5**, 09842; (*b*) A. Rödle, B. Ritschel, C. Mück-Lichtenfeld, V. Stepanenko and G. Fernández, *Chem. –Eur. J.,* **2016**, *22*, 15772–15777.

S4. S. K. Wolff, D. J. Grimwood, J. J. Mckinnon, M. J. Turner, D. Jayatilaka and M. A. Spackman, CrystalExplorer 3.1 (2013), University of Western Australia, Crawley, Western Australia, 2005-2013, http://hirshfeldsurface.net/CrystalExplorer.
